# Adsorption of Methylene Blue Dye by Calix[6]Arene-Modified Lead Sulphide (Pbs): Optimisation Using Response Surface Methodology

**DOI:** 10.3390/ijerph18020397

**Published:** 2021-01-06

**Authors:** Nor Zida Rosly, Abdul Halim Abdullah, Mazliana Ahmad Kamarudin, Siti Efliza Ashari, Shahrul Ainliah Alang Ahmad

**Affiliations:** 1 Institute of Advanced Technology (ITMA), Universiti Putra Malaysia, Serdang 43400, Malaysia; norzidarosly@gmail.com; 2 Department of Chemistry, Faculty of Science, Universiti Putra Malaysia, Serdang 43400, Malaysia; halim@upm.edu.my; 3 Department of Physics, Universiti Putra Malaysia, Serdang 43400, Malaysia; mazliana_ak@upm.edu.my; 4 Center of Foundation Studies for Agricultural Science, Faculty of Science, Universiti Putra Malaysia, Serdang 43400, Malaysia; ctefliza@upm.edu.my

**Keywords:** calix[6]arene-modified PbS, adsorption, methylene blue, isotherm, kinetic

## Abstract

Lead sulphide (PbS) modified with calix[6]arene was synthesised as an alternative and regenerative adsorbent for the adsorption of methylene blue (MB) dye. The prepared calix[6]arene-modified PbS was characterised via Fourier-transform infrared spectroscopy, field emission scanning electron microscopy, and energy-dispersive X-ray spectroscopy. The response surface methodology (RSM) based on the central composite design (CCD) was employed to identify the most significant factors, such as the initial concentration, adsorbent dosage, pH, and temperature, and to optimise the effects of the factors on the adsorptive efficiency as its response. The optimised initial concentration, adsorbent dosage, pH, and temperature were 20.00 mg/L initial concentration, 44.00 mg calix[6]arene-modified PbS, pH 6, and a temperature of 31.00 °C. A good correlation between the values and well-fitted model was observed. The adsorption performance was evaluated based on the percentage removal of MB dye from the water system. The adsorption isotherm best fit the Langmuir isotherm model, and the adsorption rate was followed by a pseudo-second-order kinetic model, a single layer chemical adsorption with a maximum adsorption capacity (q_max_) of 5.495 mg/g.

## 1. Introduction

Globally, various industries such as textile, printing, and leather industries have contributed to major sources of coloured wastewater that can cause severe water pollution [1,2]. More than 7 × 10^5^ tons/year of dyes produced using toxic, nonbiodegradable, mutagenic, and carcinogenic materials are reportedly released into the environment in wastewater without any proper treatment. Surprisingly, most of the dyes can be present in water at very minute concentrations (~1 ppm), which is sufficient to cause an aesthetic problem [3,4]. Methylene blue (MB) is one of the common aromatic–cationic dyes that are widely used in various applications, including cell staining, antimicrobial chemotherapy, phototherapy, and cancer research [5,6,7,8]. Although MB is useful in medical applications, the exposure to MB in large doses (>7.0 mg/kg) can cause harm to humans, such as skin damage, a burning sensation in the eyes, high blood pressure, digestive disease, mental confusion, nausea, abdominal pain, and methemoglobinemia [9]. Therefore, the removal of MB dye from wastewater is a critical step prior to its release into the environment.

Many technological innovations and techniques have been introduced to tackle worldwide water pollution challenges. Some common techniques are flocculation–coagulation, membrane separation, chemical and electrochemical oxidation, biodegradation, and adsorption [10,11,12,13,14,15]. Among those aforementioned techniques, adsorption is a compelling choice for the removal of dyes due to its multiple benefits, such as simplicity in design and operation, efficiency in treatment, low cost, good applicability, and high adsorption capacity [16,17]. Additionally, adsorption has been established as one of the top control techniques adopted by the United States Environmental Protection Agency (USEPA) [18].

Nanomaterials are now overtaking other types of adsorbents. They have fascinated scientists and been extensively explored to improve the efficiency and adsorption capacities of contaminant removal from wastewater. It is an ideal adsorbent due a variety of factors, such as high surface area, low cost, ready availability, and faster adsorption. Some established adsorbents in the dye adsorption process are carbon nanotubes (CNTs), graphene, ferric oxide (Fe_3_O_4_), titanium oxide (TiO_2_), magnesium oxide (MgO), manganese oxide (MnO), and zinc oxide (ZnO) [19,20,21,22]. Besides, metal sulphide-loaded nanomaterials (e.g., activated carbon) are also gaining attention as an adsorbent is due to their efficiency in wastewater treatment. However, only a few studies have explored metal sulphide modification as an alternative adsorbent in wastewater treatment systems [23]. For example, Jeong et al. synthesised a nanocrystalline mackinawite (FeS) for the removal of mercuric ions [24]. To the best of the authors’ knowledge, no study has yet analysed lead sulphide (PbS) as an adsorbent in the removal of MB dye.

Calix[n]arenes are third-generation host molecules of multifunctionalisable macromolecules after crownethers and cyclodextrins. They are formed from intrinsic pores and synthesised from formaldehyde and para-substituted phenols. They have basket-shaped hollow cavity confirmations with different ring sizes depending on the synthetic condition. Typically, these macromolecules possess a hydrophilic lower rim, mainly due to the presence of phenolic functions. On the other side, aromatic functions and aliphatic side groups that surround a hollow cavity with many dimensions depend on the specific phenolic units incorporated, which form a hydrophobic upper rim [25]. Calix[n]arene’s unique confirmations and complexation properties lend well in studies of synthetic, natural hosts, and the water treatment industry [26,27].

The efficiency of wastewater contaminant removal is influenced by several process variables. The traditional one-parameter-at-a-time approach is employed to optimise these variables. However, the main drawback of the traditional approach is that only one parameter is varied at a time, while the interactive effects among variables are not studied. Moreover, many experiments must be done using this approach, making it time-consuming and costly. Therefore, the experimental design must account for the maximum adsorption of the water effluents, helping both to reduce the cost as well as reduce the consumption of valuable resources such as energy and materials. Response surface methodology (RSM) can be carried out to optimise the effective variables while keeping a minimum number of experiments and considering the mutual interaction of the factors and their individual effects. Previous studies have demonstrated the use of RSM modelling for the adsorption of water effluents, including heavy metals, dyes, and pharmaceutical waste, with promising results [28,29].

**Figure 1:**
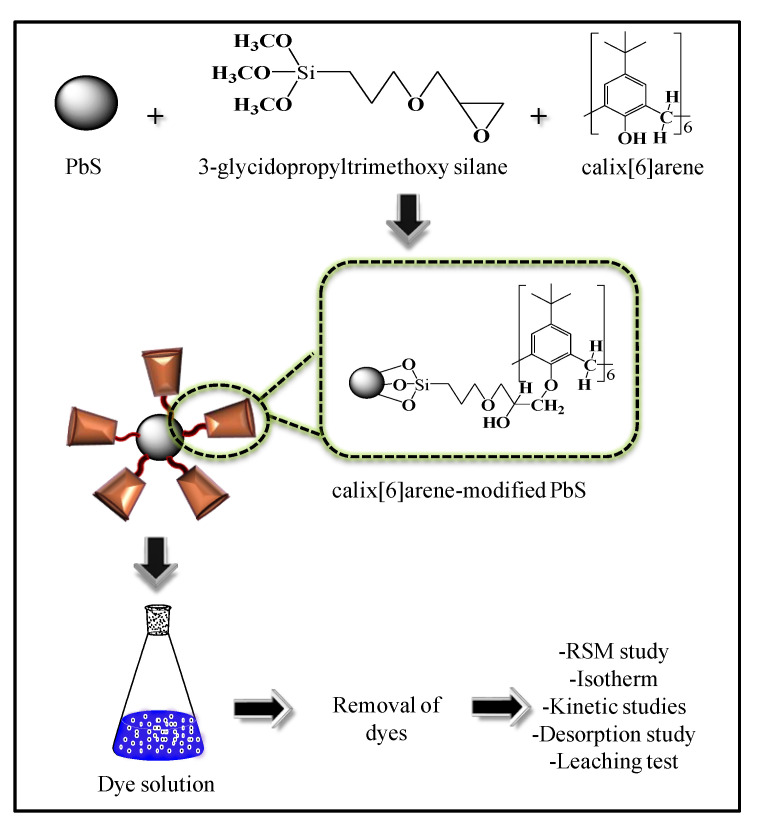
Schematic illustration of calix[6]arene-modified lead sulphide (PbS) and the methylene blue (MB) dye adsorption studies.

According to the authors’ knowledge, there is no comprehensive analysis on lead sulphide (PbS) with the host molecule, calixarene, as an adsorbent for the removal of dye wastewater. Although previous works on metal sulphide adsorbents have effectiveness in the removal of pollutants, none has described the interactive effects between the factors studied [23,24]. Indeed, the present work of metal sulphide in terms of analysis and optimisation is a novel and a new approach as compared to previous studies. This research article investigates calix[6]arene-modified PbS as an alternative nanoadsorbent for the removal of methylene blue (MB) dye. A statistical technique, RSM, is introduced to assess the actions of various independent factors on the system response. Independent variables are performed to optimise the MB adsorption to observe the adsorption capacity. Figure 1 shows the overall procedure for this study.

## 2. Experimental Works

### 2.1. Materials and Methods

Lead (II) acetate (Pb(CH_3_COO)_2_, purity, 99%), sodium hydroxide (NaOH), potassium hydroxide (KOH), hydrogen chloride (HCl, purity, 37%), toluene (C_6_H_5_CH_3_), triethylamine N(CH_2_CH_3_)_3_, hydrogen nitrate (HNO_3_, purity, 69%), ethanol (C_2_H_5_OH, purity, 95%), sodium bicarbonate (Na_2_CO_3_), sodium sulphide (Na_2_S, purity, 99%), and methylene blue (MB, purity, 99%) were purchased from R & M chemicals (Semenyih, Malaysia). Dithioglycerol (DTG), thioglycerol (TGL), and 3-glyxidoxypropyltrimethoxy silane (C_9_H_20_O_5_Si, purity, 98%) were procured from Sigma-Aldrich (Gillingham, UK). Potassium nitrate (KNO_3_) and *p-tert*-butyl-calix[6]arene (C_66_H_84_O_6_, purity, 96%) were obtained from HmbG chemicals (Hamrburg, Germany) and Alfa Aesar (Tewksbury, MA, USA), respectively.

### 2.2. Synthesis of Calix[6]Arene-Modified Lead Sulphide(Pbs)

The lead sulphide (PbS) nanoparticles were prepared based on a previous procedure, with slight modifications [30]. The precipitant of PbS was collected by mixing the solution in ethanol at a 3:1 ratio and followed by drying in an oven for 2 h at 80 °C. calix[6]arene-modified PbS was further modified by mixing 3-glycidoxypropyltrimethoxy silane (4.18 mmol), *p-tert*-butyl-calix[6]arene (3.03 mmol), and 0.2 g lead sulphide (PbS) nanoparticles with 3 drops of the catalyst, triethylamine, in 10-mL dry toluene (dried before using molecular sieves). The solution was stirred at room temperature for 6 h in an inert nitrogen gas atmosphere. The calix[6]arene-modified PbS was centrifuged and washed with toluene, ethanol, and distilled water, in that order. Lastly, the calix[6]arene-modified PbS was collected after drying at 80 °C for 3 h.

### 2.3. Characterisations

Fourier-transform infrared (FTIR) (Perkin-Elmer, Waltham, MA, USA) was used to examine the chemical functional groups of calix[6]arene-modified PbS. The samples were scanned within the region of 4000–400 cm^−1^ at room temperature. The surface morphology of the synthesised unmodified PbS and calix[6]arene-modified PbS were evaluated via field emission scanning electron microscopy (FESEM, Quanta 400F) operated in low vacuum. The elements present in calix[6]arene were determined via FESEM coupled with energy-dispersive X-ray (EDX) spectroscopy.

### 2.4. Determination of the Point of Zero Charge (PZC)

Briefly, 0.10 g of calix[6]arene-modified PbS was added to several conical flasks containing 50.0 mL of 0.01-mol/L KNO_3_ solution. The initial pH values were adjusted between pH 3 and pH 11. The pH adjustment was made using 0.10-mol/L KOH and 0.10-mol/L HNO_3_ solutions. All conical flasks were shaken for 48 h under atmospheric conditions. After centrifugation of the samples, the final pH was monitored using a pH meter (Sedolis Sartorius, Goettingen, Germany). The graph of ∆pH versus the initial pH was plotted, and the initial pH value at which ∆pH became zero was considered as the PZC of the calix[6]arene-modified PbS.

### 2.5. Adsorption Studies

The adsorption potential of calix[6]arene-modified PbS towards MB dye was determined via batch adsorption experiments. In the experiments, a standard MB dye solution of 1000 mg/L was prepared without any purification. The required dye solution concentration was achieved by diluting the stock solution appropriately with deionised water. Then, 0.1-mol/L NaOH or 0.1-mol/L HCl was used to adjust the pH of the solution. The adsorption experiments were executed in a conical flask containing 10.0-mL MB dye solution of specific concentrations. The mixture was agitated in a laboratory shaker at 150 rpm and at a contact time of 60 min. The final concentration of the sample residual dye was determined using a UV–Vis spectrophotometer (Perkin-Elmer Lambda 35, Woburn, MA, USA) at a maximum wavelength of 664 nm. The percentage removal of MB was calculated using the following Equation (1):

% MB removal = (*C_i_* − *C_f_*)/*C_i_* × 100 (1)

where *C_i_* (mg/L) is the initial concentration of MB solution before adsorption, and *C_f_* (mg/L) is the final concentration after the MB dye adsorption.

The impacts of various factors, such as the initial concentration (5–20 mg/L), adsorbent dosage (20.00–50.00 mg), initial pH (pH 3–pH 11), and temperature (27–40 °C), were examined. At the end of the experiment, the sample solution was centrifuged for 20 min at 2500 rpm.

### 2.6. Response Surface Methodology (RSM)

Response surface methodology (RSM) uses experimental designs to optimise the influence of interactive effects on the dependent variable (response). Central composite design (CCD) (Design-Expert Software version 6.0.6) is an appropriate RSM design for this research, because it recognises the effects of some independent variables on the MB adsorption of calix[6]arene-modified PbS. The MB adsorption response data from the suggested experiments was generated via the second-order polynomial equations represented by Equation (2):

(2) $$Y\;=\;\beta_{0}\;+\;\sum_{i=1}^{k}\beta_{i}x_{i}\;+\;\sum_{i=1}^{k}\beta_{ii}x^{2}\;+\sum_{i=1}^{k-1}\sum_{j>1}^{k}\beta_{ij}x_{i}x_{j}$$

where *Y* is an objective to optimise the response, $\beta_{0}$ is the constant coefficient, $\beta_{i}$ is the linear coefficient, $\beta_{ii}$ is the quadratic coefficient, and $\beta_{ij}$ is the interaction coefficient, while $x_{i}$ and $x_{j}$ represent the coded values of the independent factors.

For the purpose of selecting important variables, the parameters used for referable optimum variables were subjected to the initial concentration, adsorbent dosage, pH, and temperature and identified as A, B, C, and D, respectively. The design evaluates the individual factors and their corresponding interactions and identifies the optimum response in the minimum number of runs. The experimental error and reproducibility of the data can be gathered from the centre points. All experiments were demonstrated at a fixed contact time (60 min). The RSM/CCD suggested a total of 30 experimental runs, as presented in Table 1, including 16 factorial points, 8 axial points, and 6 centre points.

The importance of the model and the identification of the factors’ individual effects and interaction effects were evaluated using analysis of variance (ANOVA). The adequacy of the generated model was evaluated based on the (R^2^) coefficient, the adjusted coefficient of determination (adjusted R^2^), and the lack-of-fit test. Based on the *p*-values and *F*-values, the optimum conditions were predicted, and three-dimensional response–surface graphs were plotted.

### 2.7. Desorption and Reusability Tests

The reusability test of calix[6]arene-modified PbS containing MB solution was examined via a chemical desorption method. This desorption study was performed to investigate the efficiency of the adsorbent. Four different eluents (dH_2_O, HNO_3_, (CH_3_)_2_CO, and C_2_H_5_OH) were used to select the best eluent to elute the MB solutions. The adsorbent was first equilibrated with 0.025-g/L MB solution in a shaker under ambient temperature conditions. At equilibration, the adsorbent was washed with distilled water to remove unadsorbed traces of MB and then dried in an oven for 2 h at 80 °C. The various concentrations of the selected eluent were studied and tested with MB. All residual concentrations of MB were measured using UV absorbance.

### 2.8. Leaching Tests

The MB solution in each cycle was analysed to examine the amount of lead (Pb) and total suspended solids (TSS) of calix[6]arene-modified PbS. The Standard Methods (2017) 2540D were referred to determine the acceptable value for the Pb and TSS. Briefly, the supernatants were collected after the MB solution was filtered through a preweighed filter. Inductively coupled plasma-mass spectrometry (ICP-MS, Perkin Elmer, DRC-e) was used to analyse the Pb element in the MB supernatant. Meanwhile, the residue retained on the filter was heated at 104 °C until a constant weight was obtained. The concentration of suspended calix[6]arene-modified PbS was calculated using the following Equation (3):

TSS test = [final weight (mg) − original weight (mg)]/sample volume (L)(3)

## 3. Results and Discussion

### 3.1. FTIR Spectroscopic Analysis

**Figure 2:**
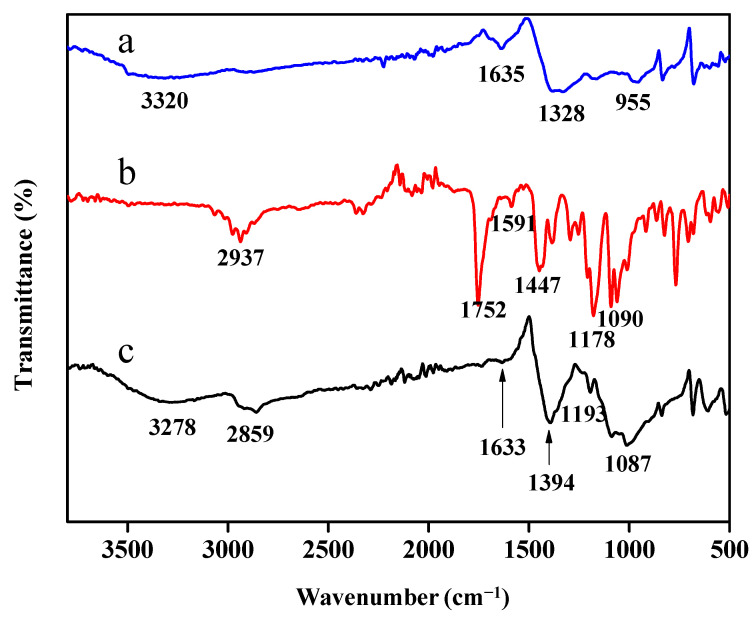
The Fourier-transform infrared (FTIR) spectra of (**a**) unmodified lead sulphide (PbS) and (**b**) *p-tert*-butyl-calix[6]arene (**c**) calix[6]arene-modified PbS.

FTIR spectroscopy was performed to understand the type of interaction obtained in the modification of calix[6]arene. According to the results (Figure 2), significant differences could be observed between the unmodified PbS and the calix[6]arene-modified PbS. The FTIR spectrum of the unmodified PbS (Figure 2a) depicts that the main feature of the spectra is the absorption band located at 3320 cm−^1^, which is assigned to the stretching vibrations of O-H groups. In Figure 2b, the main characteristic vibration of the *p-tert*-butyl calix[6]arene peaks are ascribed to the presence of the C-CH_3_ stretching vibration at 2937 cm−^1^ and the C = C aromatic vibrations at 1591 cm−^1^ and 1447 cm−^1^. Figure 2c shows the bands at 3278 cm−^1^ and 2859 cm−^1^ attributed to the OH and C-CH_3_ stretching vibrations, respectively, indicating the immobilisation of *p-tert*-butyl-calix[6]arene on the PbS surface.

### 3.2. Field Emission Scanning Electron Microscopy (FESEM) and Energy-Dispersive X-ray (EDX)

**Figure 3:**
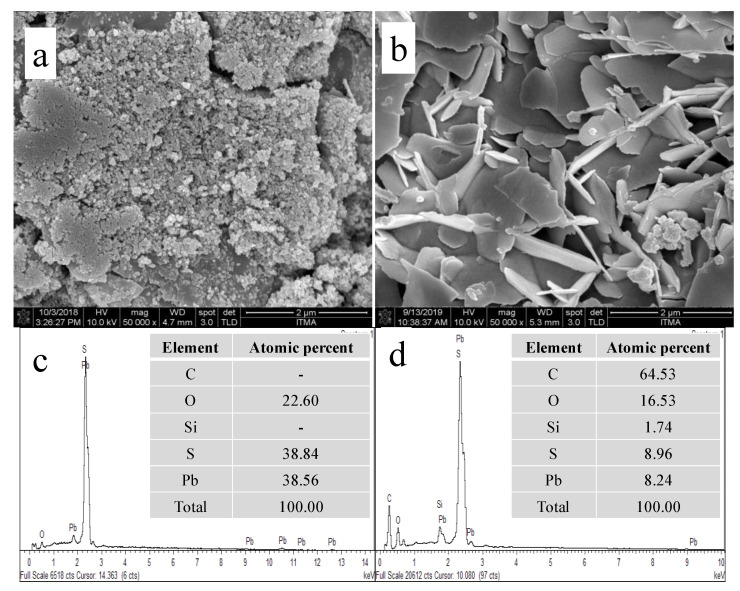
Field emission scanning electron microscopy-energy-dispersive X-ray (FESEM-EDX) analysis of the unmodified PbS (**a**,**c**) and the calix[6]arene-modified PbS (**b**,**d**).

The topological analysis and chemical compositions of the unmodified PbS and calix[6]arene-modified PbS were performed using field emission scanning electron microscopy (FESEM) and energy-dispersive X-ray (EDX). Figure 3 shows the FESEM and EDX analysis for the unmodified PbS and calix[6]arene-modified PbS. As seen in Figure 3a, the morphology of the unmodified PbS is compact and agglomerated spherical in shape, with a size smaller than 30 nm. The agglomeration of the nanoparticles is attributed to the attractive van der Waals forces between the particles [31]. Figure 3b presents the FESEM image of the calix[6]arene-modified PbS. Seen from the micrograph are nanoparticles covered by flaky materials, presumably *p-tert*-butyl calix[6]arene. The EDX spectra of both the unmodified PbS (Figure 3c) and the calix[6]arene-modified PbS (Figure 3d) clearly show well the changes that occurred after the modification of the nanoparticles. The elements displayed in the unmodified PbS (Figure 3c) mainly contain oxygen (O), sulphur (S), and lead (Pb). Meanwhile, the calix[6]arene-modified PbS indicates the presence of carbon (C) and silicon (Si) elements (Figure 3d), attributed to the effectiveness of *p-tert*-butyl calix[6]arene on the PbS surface.

### 3.3. Point of Zero Charge (PZC) of the Calix[6]Arene-Modified Pbs

**Figure 4:**
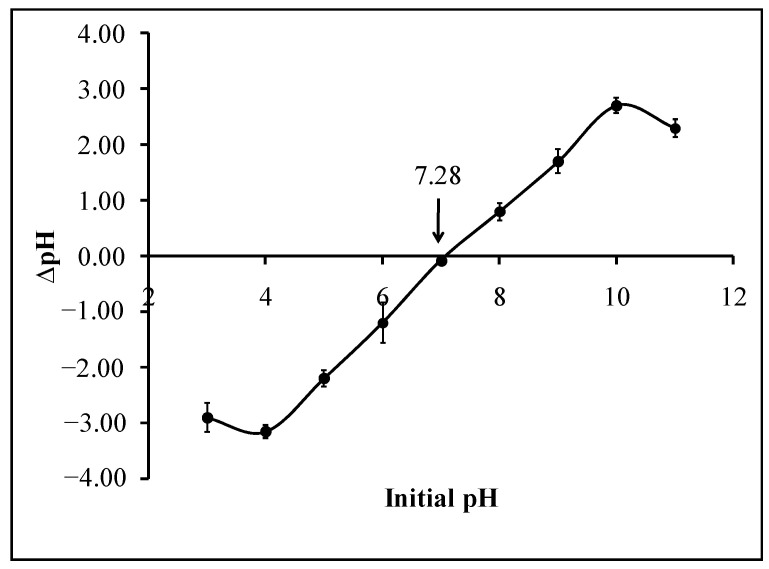
Point of zero charge (PZC) of calix[6]arene-modified PbS using KNO_3_ (0.01 M) solution under atmospheric conditions.

The point of zero charge (PZC) is the point where the density of electrical charge is zero. In this study, the solid addition method was used to determine the point of zero charge of calix[6]arene-modified PbS. In this case, pH_PZC_ represents the pH that can cause a net zero charge on the adsorbent surface. This value is significant for examining the effect of the solution pH on the adsorption process of the adsorbent material. The pH_PZC_ value of calix[6]arene-modified PbS was obtained from the intersection of ΔpH against the initial pH plot with the *x*-axis, which is pH 7.28, as shown in Figure 4. This finding suggests that calix[6]arene-modified PbS is positively charged in a solution pH lower than pH 7.28 and negatively charged in a solution pH higher than pH 7.28. The suspension of the adsorbent in the KNO_3_ solution creates an electrical charge that is due to the dissociation of the surface hydroxyl groups and the complexation of the background electrolyte ions. Briefly, for a solution with a pH lower than the PZC value, the adsorption of the protons increased, and the adsorbent behaves as anion exchangers, which have a predomina of positive charges on the surfaces. However, when the pH is above the PZC value, desorption of the protons occurs, and the surface bears as a cation exchanger, which has a net negative charge [32].

### 3.4. Model Fitting via RSM

The modelling and optimisation of the initial concentration, adsorbent dosage, pH, and temperature by RSM/CCD were employed to maximise the percentage adsorption of MB. The RSM resulted in a regression equation that connects the MB adsorption to the variables, per Equation (4) below (regarding coded factors):

(4) $$\mathrm{Adsorption}\;=\;81.69\;+\;0.21\;A\;+3.601\;B\;+4.21\;C\;-4.454\;D\;+\;2.65{\;A}^{2}\;-6.75{\;B}^{2}\;+\;2.07{\;C}^{2}\;-5.20{\;D}^{2}\;+\;2.70\;\mathrm{AB}\;+\;0.781\;\mathrm{AC}\;+1.11\;\mathrm{AD}\;-\;1.62\;\mathrm{BC}\;-\;0.52\;\mathrm{BD}\;+\;0.12\;\mathrm{CD}$$

where A is the initial concentration, B is the adsorbent dosage, C is the pH, and D is the temperature of the MB solution.

An analysis of variance (ANOVA) was demonstrated to assess the statistical significance of the quadratic model, with the analysis results presented in Table 2. The predicted values are quite close to the experimental values, thereby suggesting that the quadratic regression model is the best-fit model for the adsorption of MB. Previous studies have reported a more noteworthy effect on the response if a high *F*-value and a low *p*-value (<0.05) are obtained [33]. According to the results in Table 2, the *F*-value regression of the quadratic model is 338.5, and a very low *p*-value (<0.0001) is obtained. This result suggests that the quadratic model is statistically significant, and the model can explain most of the variations in the response. Meanwhile, the *F*-value of 4.47 from the lack of fit indicates an insignificant lack of fit that is relative to pure error, as the *p*-value prob > *F*-value is 0.0561, which is greater than 0.1000.

The coefficient of determination (R^2^) can explain the overall efficiency of a model’s prediction. The R^2^ identifies the total variation of the predicted or the model values from the mean. For a model with good prediction efficiency, the value of R^2^ should be close to 1.0. Table 2 shows the proposed model having a relatively high coefficient of determination, R^2^ (0.9968), indicating that the regression model can explain most of the variation in the data. The model could not clarify only 0.32% of the variations. Otherwise, the closeness between the high adjusted R^2^ (0.9939) value and the predicted R^2^ (0.9832) value confirms the reliability of the model in turn, supporting the high significance of the model. The prediction error sum of squares (PRESS) measures the predictive ability of the model, with the small PRESS (25.99) value indicating a desirable model. The *p*-values determine the significance of all the linear factors (A, B, C, and D) and the interactions between each; *p*-values lower than 0.05 imply that the model significance is at a 95% confidence interval. Based on the results in Table 2, the *p*-values for the linear factors B, C, and D and their interactions with the other factors are lower than 0.05, indicating significance towards the adsorption of MB.

**Figure 5:**
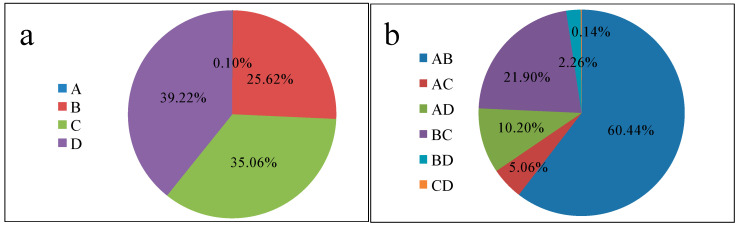
Percentage contributions of the (**a**) individual factors (A, B, C, and D) and (**b**) interacting factors on the adsorption of methyele blue (MB).

The percentage contributions of the individual factors are presented in Figure 5a. The order of the affecting factors are D > C > B > A, demonstrating that D, C, and B primarily influence the adsorption of MB, while A has the lowest percentage contribution (0.01%) and can be negligible for the adsorption of MB, as evidenced by its *p*-value (0.1395). Meanwhile, for the interacting factors, the interaction between A and B (initial concentration and adsorbent dosage) were the most significant factors, with a 60.44% contribution. Figure 5b shows the percentage contributions of the interacting factors towards the optimal adsorption of MB.

**Figure 6:**
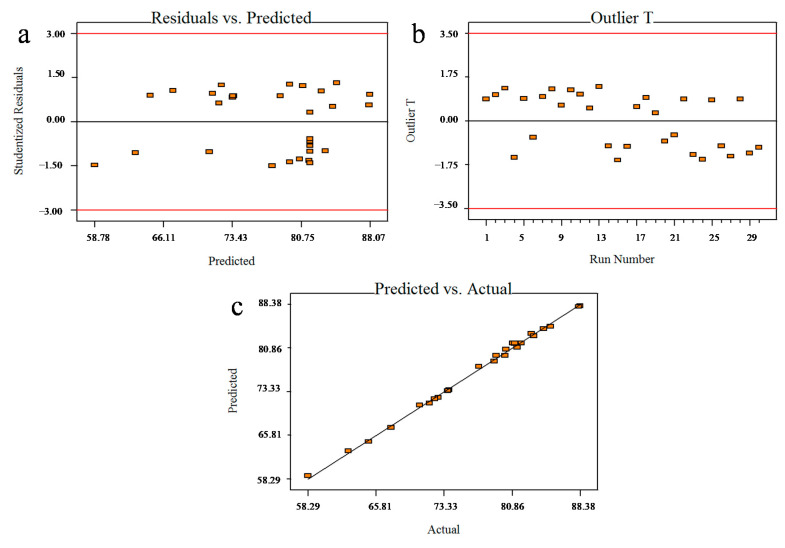
Plots of MB uptake by calix[6]arene-modified PbS (**a**) residual versus predicted, (**b**) outlier versus run number, and (**c**) predicted versus actual.

The plot of studentised residuals against the predicted response was used to evaluate the adequacy of the model. The plot in Figure 6a presents a uniformly circulated data around the mean point of the response variables. Hence, the model can be classified as adequate. The plot of outliers versus the run number in Figure 6b shows that all points are distributed within limits, indicating that all the data in this study is acceptable. Additionally, the plot of the predicted versus actual values in Figure 6c displays a good correlation between both adsorption values, indicating that the data plotted are highly accurate. Based on the results, this model is an acceptable adequate correlation model, so it can be used to represent the experimental data.

### 3.5. Interactive Effects of Factors Involved in the Adsorption of MB

**Figure 7:**
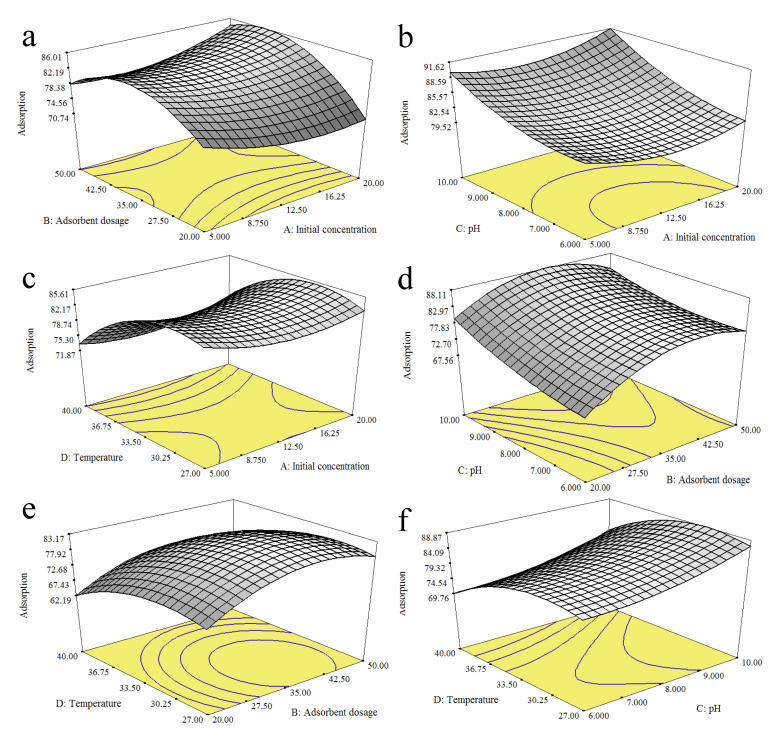
Response surface plots of the interactive effects of the (**a**) initial concentration and adsorbent dosage, (**b**) initial concentration and pH, (**c**) initial concentration and temperature, (**d**) adsorbent dosage and pH, (**e**) adsorbent dosage and temperature, and (**f**) pH and temperature.

The graphical representations of the response surfaces are illustrated based on the quadratic regression Equation (4). The 3D response surface plots describe the major effects of each factor on the response and other factors. These graphs are illustrated Figure 7a–f based on the interactions of the initial concentration (A)–adsorbent dosage (B), initial concentration (A)–pH (C), initial concentration (A)–temperature (D), adsorbent dosage (B)–pH (C), adsorbent dosage (B)–temperature (D), and pH (C)–temperature (D). These plots imply that the percentage adsorption of MB increases with the increasing initial concentration, from 5.00 mg/L to 20.00 mg. The increase in the percentage adsorption of MB is due to the available active sites on the calix[6]arene-modified PbS surface. Meanwhile, for the adsorbent dosage, the increased percentage adsorption was observed from 20.00 mg to 44.00 mg. This increase corresponds to the increase in the surface area of available active sites of the adsorbent for interactions with the MB solution. A further addition of the adsorbent caused a gradual decrease or a cease in the percentage adsorption, possibly due to the partial overlapping of calix[6]arene-modified PbS, thereby leading to the saturation of binding sites and a decrease in the effective surface area available for adsorption. The percentage adsorption increases with increasing the pH from pH 6 to pH 10. In an acidic condition (pH 6), surface repulsion occurs between the acidic phenolic hydroxyl (+OH_2_) groups of the calix[6]arene moiety in calix[6]arene-modified PbS and the MB dye (cationic dye):

$$\mathrm{Calix}\left[6\right]-\mathrm{OH}+H_{3}O^{+}\to \mathrm{Calix}\left[6\right]-{\mathrm{OH}}_{2}^{+}+H_{2}O$$

where Calix[6] denotes the surface of calix[6]arene-modified PbS.

The repulsion of the negatively charged calix[6]arene-modified PbS surface and the MB dye may be expressed as:

$$\mathrm{Calix}\left[6\right]-{\mathrm{OH}}_{2}^{+}+{\mathrm{MB}}^{+}\to \mathrm{Calix}\left[6\right]-{\mathrm{OH}}_{2}^{+}/{\mathrm{MB}}^{+}$$

where MB^+^ denotes the cationic MB dye.

The adsorption is higher at high pH due to the attraction between the negatively charged calix[6]arene-modified PbS surface and the cationic dye [32]:

$$\mathrm{Calix}\left[6\right]-\mathrm{OH}+{\mathrm{OH}}^{-}\to \mathrm{Calix}\left[6\right]-O^{-}+H_{2}O$$

The attraction of the negatively charged calix[6]arene-modified PbS surface and the MB dye may be expressed as

$$\mathrm{Calix}\left[6\right]-O^{-}+{\mathrm{MB}}^{+}\to \mathrm{Calix}\left[6\right]-O^{-}{\mathrm{MB}}^{+}$$

Based on the temperature study, the MB adsorption was observed to increase from 27 °C to 31 °C. An increase in temperature could cause the breakdown of internal bonds within the active sites of the adsorbent, thus facilitating higher MB adsorption. This fact is supported by previous studies that showed that an increase in temperature promoted stronger adsorbate molecules, which reduced the solution viscosity through the scatter rate across the external limit layer and the internal pores of the adsorbent particles [34]. However, a further increase in the temperature caused a gradual decrease in the percentage adsorption. This result may correspond to an increase in the solubility of the MB dye, resulting in a stronger interaction force between the dye and the solvent as compared to the dye and calix[6]arene-modified PbS. A decrease in the adsorption of the MB dye is also related to the increased Brownian movement of the molecules in the solution [35].

The model was validated by conducting three sets of experiments generated from the CCD-RSM, and the predicted and experimental values were compared. The validation process was done by inserting the desirable value, shown in Table 3. The predicted and experimental values from the optimised percentage adsorption of MB indicate that all the adsorption experiments have a response of less than 2% residual standard error (RSE), thus validating the model. Based on the performed experiments, the prediction of this model is up to 98% accurate. During the optimisation step, the percentage adsorption of MB was set to “maximise” with maximum importance. The pH factor was set to “minimise”, because the prepared MB was around pH 6 (minimum pH). The intent was to minimise the extraneous addition of acid/alkali while the other factors were kept within the studied range. The optimised values of the initial concentration of the MB solution, the adsorbent dosage of the calix[6]arene-modified PbS, pH, and temperature, were found to be 20.00 mg/L, 44.00 mg, pH 6, and 31.00 °C, respectively.

### 3.6. Process Modelling

#### 3.6.1. Adsorption Isotherm Evaluation

Adsorption studies are performed to determine the adsorption mechanisms involved in a given system. Besides, it also allows researchers to examine the theoretical maximum adsorption capacity of an adsorbed dye and an adsorbent. To study the relationship between the adsorbed dye and the adsorbent, various models were tested. The most common linear models are the Langmuir and Freundlich isotherms [36]. The Langmuir model presumes that the adsorption process is homogenous within a single layer, involving a limited number of identical active sites. Meanwhile, Freundlich is an empirical isotherm that assumes a multilayer adsorption process on a heterogeneous surface. The Langmuir and Freundlich isotherm models are expressed by Equation (5) and Equation (6), respectively.

Langmuir equation:

(5) $$\frac{C_{e}}{q_{e}}=\frac{1}{K_{L}q_{\max}}+\frac{1}{q_{\max}}C_{e}$$

Freundlich equation:

(6) $${\log\;q}_{e}\;={\;\log\;K}_{F}+\frac{1}{n}{\log\;C}_{e}$$

where C_e_ (mg/L) and q_e_ (mg/g) are the concentration and the adsorption capacity at the equilibrium condition, respectively. The constant K_L_ (L/mg) related to free energy and q_max_ (mg/g), the maximum adsorption capacity, are derived from the intercept and the slope of the linear plot of Equation (5). Meanwhile, K_F_ (L/mg) and 1/n are the Freundlich constant and the heterogeneity factor, respectively. Both are derived from plotting the log q_e_ versus log C_e_ from Equation (6).

**Figure 8:**
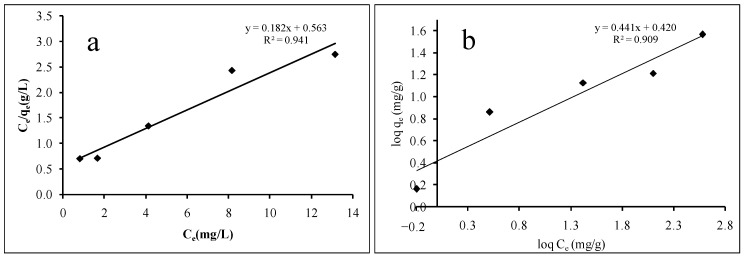
Linear plot of (**a**) the Langmuir isotherm and (**b**) the Freundlich isotherm of MB adsorption at 44.00 mg of calix[6]arene-modified PbS, pH 6, and 31.00 °C.

Figure 8a,b illustrate the Langmuir and Freundlich adsorption isotherms, respectively. The isotherm model constants were calculated via the linear regression method and are listed in Table 4. The results show that the equilibrium adsorption of MB by calix[6]arene-modified PbS is described better by the Langmuir isotherm (R^2^ = 0.941) compared to the Freundlich isotherm (R^2^ = 0.909). This result suggests that the adsorption of MB ions probably occurs as a single layer on adsorption sites that are homogenously distributed onto calix[6]arene-modified PbS, wherein all molecules are predicted to have uniform activation energy without any interaction between the adsorbed ions.

The isotherm model constants, calculated by employing the linear regression method, are listed in Table 4. A critical parameter in the Langmuir equation is the value of the equilibrium dimensionless parameter R_L_ (separation factor), which is expressed [37] by the following Equation (7):

R_L_ = 1/(1 + K_L_ C_o_) (7)

R_L_ describes the isotherm shape and predicts the feasibility of a sorption system, whether favourable (0 < R_L_ < 1) or non favourable (R_L_ > 1). In this study, the R_L_ values at 5, 10, 15, 20, and 30 mg/L were found to be 0.3821, 0.2362, 0.1710, 0.1339, and 0.0935, respectively. The R_L_ values for all MB concentrations fell in-between 0 and 1, confirming the favourability of the MB adsorption by calix[6]arene-modified PbS. Moreover, the R_L_ results display a decrease in values when the initial concentration of MB was increased due to the favourable adsorption of calix[6]arene-modified PbS at higher concentrations. The Freundlich constant “n” gives information on the favourability of the adsorption process. If the value of “n” exceeds 1, the adsorbate is easy to adsorb, and if the value is lower than 0.5, the adsorbate is difficult to adsorb [38]. According to the result in Table 4, the value of “n” (2.268) was found to be greater than 1, implying that the Freundlich isotherm model is equally applicable for the adsorption of MB dye.

The maximum adsorption capacities (q_max_) of calix[6]arene-modified PbS was compared with other potential adsorbents and listed in Table 5. The table shows that the maximum adsorption capacity of calix[6]arene-modified PbS (5.495 mg/g) is lower than some of the reported adsorbents. However, the RSM technique is an important optimisation process that managed to include the interactive effects of the factors studied.

#### 3.6.2. Adsorption Kinetic Modelling

Adsorption kinetic modelling provides insight into the rate of MB dye travel from a liquid to a solid system and the potential rate-controlling step. Pseudo-first-order and pseudo-second-order kinetic models were thus employed to evaluate the adsorption behaviours, such as the rate of adsorption and the nature of interactions between the MB molecules and the calix[6]arene-modified PbS surface. The best kinetic model was evaluated based on the and the adsorption capacity values q_e1,cal_ and q_e2,cal_.

The pseudo-first-order model is expressed by Equation (8):

(8) $$\ln\left(q_{e}-q_{t}\right)={\mathrm{lnq}}_{e}-k_{1}t$$

where q_e_ and q_t_ are the adsorption capacities at equilibrium and time, respectively. k_1_ is the rate constant of pseudo first-order model.

The values of k_1_ and q_el,cal_ were calculated from the slope and the intercept of the linear graph of ln(q_e_ − q_t_ ) versus t.

The pseudo-second-order equation is expressed as Equation (9):

(9) $$\frac{t}{q_{t}}=\frac{1}{k_{2}q_{e}2}+\frac{1}{q_{e}}t$$

in which k_2_ is the rate constant of pseudo second-order model.

To obtain the values of k_2_ and q_e2,cal_, a linear graph of $\frac{t}{q_{t}}$ versus t was plotted and its slope and intercept calculated.

Then, the values of the two models were compared, calculated, and compiled in Table 6. The results show that, for the five different concentrations of MB dye, the model that fitted well to the experimental data was the pseudo-second-order model, which showed the highest value of the linear regression coefficients (R^2^) and a good agreement between the calculated q_e2,cal_ and the experimental q_e,exp_ values. Table 6 summarises the q_e,cal_ of the two kinetic models, with the MB concentrations ranging from 5 ppm to 30 ppm. The R^2^ value of the pseudo-second-order kinetic model was larger than 0.975 at different initial concentrations, as compared to the R^2^ of the pseudo-first-order kinetic model (0.782). As seen in Table 6, the k_2_ values of the pseudo-second-order decreased when the initial concentration of the MB dye increased. This might be attributed to the higher levels of MB ions competing for the limited adsorption active sites.

**Figure 9:**
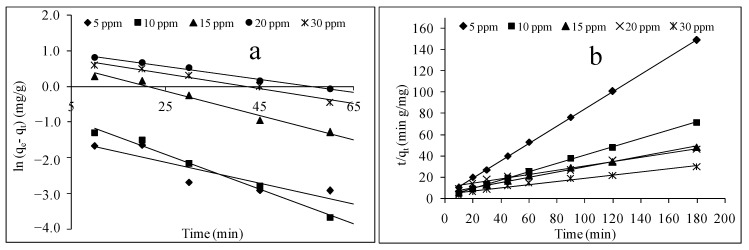
Pseudo-first-order kinetic (**a**) and pseudo-second-order kinetic (**b**) models of MB adsorption by calix[6]arene-modified PbS at 44.00 mg of calix[6]arene-modified PbS, pH 6, and 31.00 °C.

The curve-fitting plots of the MB adsorption by calix[6]arene-modified PbS for both kinetic models are presented in Figure 9. The results show that the curve-fitted plots of $\frac{t}{q_{t}}$ versus t (Figure 9b) of the second-order kinetic model displayed an acceptable fit, while the first-order kinetic model plots of ln ($q_{e}$ − $q_{t}$) against t (Figure 9a) did not have a good fit with the experimental values. These observations suggest that the rate-limiting step on the system may be a chemical sorption process that involves valence forces through the sharing or exchange of electrons between the calix[6] arene-modified PbS and MB molecules.

### 3.7. Proposed Mechanism of Adsorption

**Figure 10:**
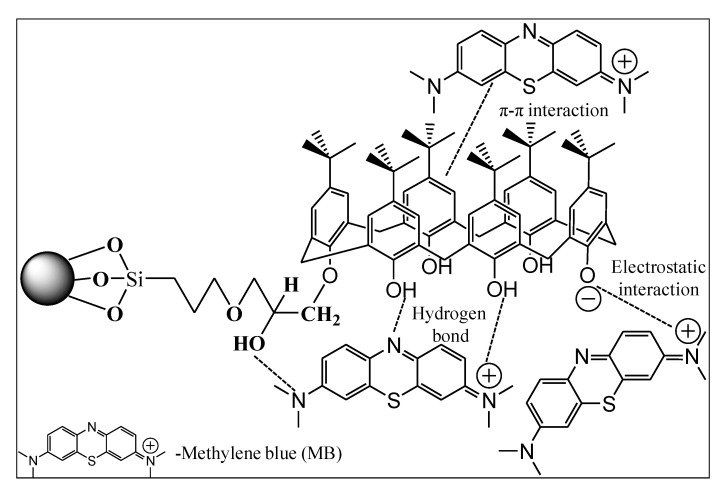
Schematic illustration of the mechanism of MB adsorption by calix[6]arene-modified PbS.

The adsorption process is generally related to the nature of the adsorbate and its performance. It also depends on factors such as the specific surface area, organic macromolecular steric hindrance, heterogeneous adsorption sites, and the interaction between adsorbent and adsorbate, as well as the surface charge of the adsorbent [48,49]. The surface charge of calix[6]arene-modified PbS is highly affected by the pH. The pH at the point of zero charge (pH_pzc_) was pH 7.28. When the pH of the solution is above the pH_pzc_ (pH > 7.28), the surface of calix[6]arene-modified PbS acquires a negative charge via the deprotonation of the exposed hydroxyl groups. This process involves the electrostatic interaction between the MB dye (cationic dye) and the surface of calix[6]arene-modified PbS, as proposed in Figure 10. The adsorption mechanism, such as the hydrogen bond interaction, is suggested between the hydroxyl group (O-H) available on the surface of the adsorbent and the nitrogen (N) atom in the MB dye structure. Another possibility is that these functional groups can form a π-π interaction between the calix[6]arene-modified PbS and the MB dye. According to the adsorption mechanisms, it can be concluded that these interactions are effective at enhancing the MB dye removal by calix[6]arene-modified PbS.

### 3.8. Desorption and Reusability Tests

Stability and reusability are important aspects of an ideal adsorbent, which help reduce the cost of treatment procedures and adsorbent supply and help minimise the adsorbent disposal problem. The reusability test of calix[6]arene-modified PbS was done by evaluating the MB adsorption efficiency after treatment with four different eluents (dH_2_O, HNO_3_, (CH_3_)_2_CO, and C_2_H_5_OH).

**Figure 11:**
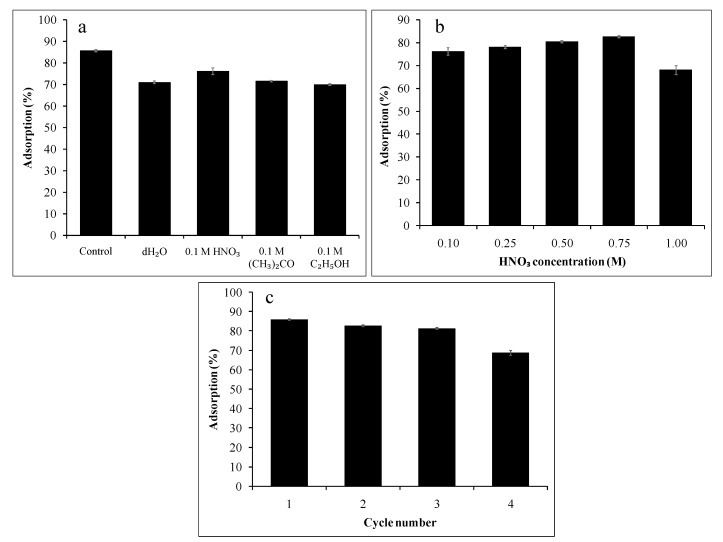
The adsorption of MB by calix[6]arene-modified PbS (**a**) after treatment with various types of desorption eluents and (**b**) with different HNO_3_ concentrations and (**c**) reusability tests at 20.0 mg/L of MB dye, 44.00 mg of calix[6]arene-modified PbS, pH 6, and 31.00 °C.

The effectiveness of these different eluents is presented in Figure 11a. As can be seen, the maximum percentage adsorption of MB before the desorption process was 85.80% (control). After treatment with the different eluents, the HNO_3_ and C_2_H_5_OH solutions were found to produce the highest (78.15%) and the lowest (70.07%) adsorptions of MB dye, respectively. The high reusability of the HNO_3_ solution can be explained by the physical nature of the interactions between calix[6]arene-modified PbS and the MB dye. At low pH, the protonation of the adsorbent causes an electrostatic repulsion between the positively charged sites on the calix[6]arene-modified PbS and the cationic MB dye molecules. The second reason might be the abundance of hydrogen ions (H^+^) in the acidic solution that can be exchanged with the MB ions on calix[6]arene-modified PbS [50]. Thus, the desorbing agents that can produce more cations in the solution—especially, H^+^—are selected for the desorption of the cationic dye.

Next, the effects of different concentrations of the HNO_3_ eluent, ranging from 0.10 to 1.00 M, were investigated on the loaded MB with calix[6]arene-modified PbS. Figure 11b illustrates the adsorption of MB by calix[6]arene-modified PbS after treatment with different concentrations of HNO_3_. The adsorption of MB increased after the treatment based on the increased eluent concentration, ranging from 0.10 to 0.75 M. The highest amount of desorption (82.75%) was observed when 0.75-M HNO_3_ was used as the eluent. Increasing the HNO_3_ concentration resulted in an increased H+ ion concentration, causing more dye to desorb from calix[6]arene-modified PbS. However, the adsorption of MB slightly dropped after the calix[6]arene-modified PbS was treated with 1.00-M HNO_3_. Meanwhile, Figure 11c demonstrates the cyclic adsorption–regeneration test of the calix[6]arene-modified PbS. After four cycles, the adsorbed percentage of MB markedly decreased from 85.80% to 68.72%.

### 3.9. Leaching Tests of Calix[6]Arene-Modified Pbs

To evaluate the stability of calix[6]arene-modified PbS and to ensure quality control of the wastewater treatment, leaching tests were carried out. The TSS test measured all particles in the water that did not pass through the filter after the adsorption process. According to the compounds listed in the Malaysian Environmental Quality (Industrial Effluent) Regulations 2009 [51], the acceptable discharge values must be lower than 0.50 mg/L and 50 mg/L for Pb and the suspended solids, respectively. The ICP-MS results of the leaching tests of the Pb results and the TSS values of four different cycle numbers of calix[6]arene-modified PbS are shown in Table 7. The values are acceptable, since they are lower than the limit values, thus suggesting the high stability of the adsorbent synthesised in the present research.

## 4. Conclusions

The current paper demonstrated the synthesis of calix[6]arene-modified PbS and its efficient cationic dye adsorption. The effects of the initial concentration, adsorbent dosage, pH, and temperature on the adsorption were studied using the response surface methodology (RSM) called central composite design (CCD). Equilibrium isotherm and kinetic studies were carried out under optimised conditions. The optimised initial concentration, adsorbent dosage, pH, and temperature were 20.00 mg/L of MB dye, 44.00 mg of calix[4]arene-modified PbS, pH 6, and 31.00 °C temperature. The adsorption of the MB dye was in-line with the Langmuir isotherm attributed to the homogenous monolayer adsorption that occurred on the surface of calix[6]arene-modified PbS. The maximum adsorption capacity of calix[6]arene-modified PbS was found to be 5.495 mg/g at the optimal conditions. The kinetic data closely followed the pseudo-second-order kinetic model, indicating chemisorptions as the mechanism of adsorption.

## Figures and Tables

**Table 1:** The 4-factor central composite design (CCD) matrix and the value of the response function methylene blue (MB) (%).

Run	Factors				Response Adsorption (%)	
	A. Initial Concentration (mg/ L)	B: Adsorbent Dosage (mg)	C: pH	D: Temperature	Actual	Predicted
1	20.00	50.00	6	40.00	73.89	73.60
2	5.00	50.00	10	27.00	83.25	82.90
3	12.50	35.00	6	33.50	80.07	79.55
4	12.50	35.00	8	33.50	80.92	81.69
5	20.00	20.00	6	27.00	64.98	64.68
6	12.50	35.00	8	33.50	81.32	81.69
7	12.50	20.00	8	33.50	71.72	71.33
8	20.00	20.00	10	40.00	72.70	72.28
9	12.50	35.00	8	40.00	72.28	72.02
10	12.50	35.00	8	27.00	81.43	80.93
11	5.00	50.00	6	40.00	67.47	67.12
12	5.00	35.00	8	33.50	84.34	84.13
13	20.00	35.00	8	33.50	85.09	84.55
14	12.50	35.00	8	33.50	81.14	81.69
15	20.00	20.00	10	27.00	77.17	77.67
16	5.00	50.00	10	40.00	70.64	70.98
17	12.50	35.00	10	33.50	88.21	87.98
18	20.00	50.00	10	27.00	88.38	88.07
19	12.50	35.00	8	33.50	81.86	81.69
20	12.50	35.00	8	33.50	81.24	81.69
21	12.50	35.00	8	33.50	81.37	81.69
22	12.50	50.00	8	33.50	78.89	78.53
23	20.00	50.00	6	27.00	81.15	81.59
24	20.00	20.00	6	40.00	58.29	58.78
25	5.00	20.00	6	27.00	73.73	73.45
26	5.00	20.00	10	27.00	82.98	83.31
27	5.00	50.00	6	27.00	79.09	79.55
28	5.00	20.00	10	40.00	73.78	73.49
29	20.00	50.00	10	40.00	80.17	80.59
30	5.00	20.00	6	40.00	62.76	63.11

**Table 2:** Analysis of variance (ANOVA) of the response surface quadratic model for the MB adsorption of calix[6]arene-modified PbS. R^2^: coefficient of determination.

Variation Source	Coefficients	Sum of Squares	Degree of Freedom	Mean Square	*F*-Value	*p*-Value Prob > *F*
Intercept	81.69					
Linear						
A	0.21	0.7938	1	0.7938	2.435	0.1395
B	3.60	233.4	1	233.4	716.0	<0.0001
C	4.21	319.6	1	319.6	980.4	<0.0001
D	−4.45	357.2	1	357.2	1096	<0.0001
Quadratic						
A^2^	2.65	18.22	1	18.22	55.90	<0.0001
B^2^	−6.75	118.3	1	118.3	362.9	<0.0001
C^2^	2.07	11.18	1	11.18	34.29	<0.0001
D^2^	−5.20	70.27	1	70.27	215.5	<0.0001
Interaction						
AB	2.70	116.9	1	116.9	358.6	<0.0001
AC	0.78	9.781	1	9.781	30.00	<0.0001
AD	1.11	19.74	1	19.74	60.54	< 0.0001
BC	−1.62	42.35	1	42.35	129.9	< 0.0001
BD	−0.52	4.379	1	4.379	13.34	0.0023
CD	0.12	0.2627	1	0.2627	0.8057	0.3836
Model		1545	14	110.4	338.5	<0.0001
Residual		4.89	15	0.3260		
Lack-of-fit		4.398	10	0.4398	4.469	0.0561
PRESS = 25.99						
R^2^ = 0.9968						
Adjusted R^2^ = 0.9939						
Predicted R^2^ = 0.9832						

**Table 3:** The predicted and experimental values of the optimum combination factors. RSE: residual standard error.

					Adsorption (%)		
Number	Initial Concentration (mg/L)	Adsorbent Dosage (mg)	pH	Temperature (°C)	Predicted	Experiment	RSE (%)
1	20.0	43.70	6	30.72	84.64	85.80	1.37
2	5.00	37.55	6	29.71	84.60	83.12	1.75
3	20.0	41.20	6	30.62	84.36	85.33	1.15

**Table 4:** The Langmuir and Freundlich isotherm parameters and regression data for the adsorption of MB onto calix[6]arene-modified PbS at 44.00 mg of calix[6]arene-modified PbS, pH 6, and 31.00 °C.

Isotherm	Parameters	Values
Langmuir	q_max_	5.495 mg/g
	K_L_	0.3233 L/mg
	R^2^	0.941
Freundlich	K_F_	2.630 L/mg
	N	2.268
	R^2^	0.909

**Table 5:** Comparison of the maximum adsorption capacity of MB with various adsorbents. RSM: response surface methodology.

Adsorbent for MB	Maximum Adsorption Capacity (mg/g)	Type of Optimisation Method	References
Coir pith carbon	5.870	One-factor-at-a-time	[39]
Apricot stones-carbon	4.110	One-factor-at-a-time	[40]
Zeolite	8.670	One-factor-at-a-time	[41]
CuO-NP-AC	10.550	One-factor-at-a-time	[42]
MgO	9.270	One-factor-at-a-time	[43]
Fe_3_O_4_@GTPs NPs	7.250	One-factor-at-a-time	[44]
Peanut stick activated carbon	2.570	One-factor-at-a-time	[45]
Natural Chinese zeolite	5.150	One-factor-at-a-time	[46]
Wood millet carbon	3.745–4.739	RSM	[47]
calix[6]arene-modified PbS	5.495	RSM	This work

**Table 6:** Kinetic study of MB adsorption by calix[6]arene-modified PbS at 44.00 mg of calix[6]arene-modified PbS, pH 6, and 31.00 °C.

C_0_ (mg/L)	q_e,exp_ (mg/g)	Pseudo-First-Order			Pseudo-Second-Order		
		q_e1,cal_ (mg/g)	k_1_ (min^−1^)	R^2^	q_e2,cal_ (mg/g)	k_2_ (min^−1^)	R^2^
5	1.179	0.2481	0.020	0.782	1.232	0.199	0.999
10	2.366	1.966	0.018	0.987	2.558	0.091	0.999
15	3.087	2.065	0.034	0.976	4.082	0.011	0.992
20	3.362	2.779	0.048	0.991	4.926	0.004	0.975
30	4.788	2.401	0.029	0.971	6.667	0.006	0.978

where C_0_ is the initial concentration of MB.

**Table 7:** The inductively coupled plasma-mass spectrometry (ICP-MS) results of the leaching test of Pb and the total suspended solids (TSS) test in calix[6]arene-modified PbS at different cycle numbers at 20.0 mg/L of MB dye, 44.00 mg of calix[6]arene-modified PbS, pH 6, and 31.00 °C.

Cycle Number	1	2	3	4
Pb element (mg/L)	0.052 ± 0.02	0.1514 ± 0.01	0.2857 ± 0.03	0.3401 ± 0.05
TSS test (mg/L)	13.27 ± 2.11	15.16 ± 2.47	15.78 ± 2.32	16.02 ± 2.02

## Data Availability

Data sharing not applicable.
